# Supplementary material for: Magnesium galvanic cells produce hydrogen and modulate the tumor microenvironment to inhibit cancer growth
Source: Nat Commun. 2022 Apr 28;13:2336. doi: 10.1038/s41467-022-29938-6 (PMC9051066; doi:10.1038/s41467-022-29938-6)
Supplement: Supplementary file 2 — Supplementary Information [file 41467_2022_29938_MOESM2_ESM.pdf]

Supporting Information

Magnesium galvanic cells produce hydrogen and modulate the tumor microenvironment to inhibit cancer growth

Nailin Yang[1], Fei Gong[1], Bo Liu[1], Yu Hao[1], Yu Chao[1], Huali Lei[1], Xiaoyuan Yang[1], Yuehan Gong[1], Xianwen Wang[1], Zhuang Liu[1]*, and Liang Cheng[1]*

[1]Institute of Functional Nano & Soft Materials (FUNSOM), Jiangsu Key Laboratory for Carbon-Based Functional Materials and Devices, Soochow University, Suzhou 215123, China.

*Corresponding authors: Email: lcheng2@suda.edu.cn; zliu@suda.edu.cn

Supplementary **Fig. 1-2** H_2 generation in 24 h from MgG rods.

Supplementary **Fig. 3** SEM and EDS mapping images of Mg rods.

Supplementary **Fig. 4** EDS spectra of Mg and MgG.

Supplementary **Fig. 5** Characterization of Pt NPs.

Supplementary **Fig. 6** XRD sprectrum of Mg rods.

Supplementary **Fig. 7-8** Time-dependent absorption spectra of the MB solution via various treatments.

Supplementary **Fig. 9** O_2 content changes after the reaction between Mg or MgG rods and H_2O.

Supplementary **Fig. 10** XPS characterization of MgG before and after H_2 generation process.

Supplementary **Fig. 11** Quantitative analysis of the fluorescence intensity of JC-1 monomers in 4T1 cells.

Supplementary **Fig. 12** Flow cytometry data to show DCF-positive CT26 cells after different treatments.

Supplementary **Fig. 13** Relative cell viabilities of 4T1 and CT26 cells after incubation with various Mg^{2+} concentrations for 24 h.

Supplementary **Fig. 14** H_2 generation performance of MgG rods and different kinds of the commercialized Mg alloys.

Supplementary **Fig. 15** Relative viabilities of 4T1 cells after various treatments.

Supplementary **Fig. 16** Cell viabilities of 4T1 and CT26 cells cultured with the pure H_2.

Supplementary **Fig. 17** Flow cytometry analysis of CT26 cells after various treatments.

Supplementary **Fig. 18** Schematic illustration the position of Mg or MgG rods in the tumors of mice or rabbits implanted by a simple implant approach.

Supplementary **Fig. 19** In vivo time-dependent ultrasonic imaging of 4T1 tumor-bearing mice post i.t. implanted with Mg rods.

Supplementary **Fig. 20-21** SEM image and EDS element mapping of MgG rods, and H_2 generation from MgG rods in PBS solutions after implantation into the tumor for 4 h and 24 h.

Supplementary **Fig. 22** The infrared thermal images and temperature change curves of mice tumor.

Supplementary **Fig. 23-24** Flow cytometry gating strategy and flow cytometric analysis results of different cells in tumor.

Supplementary **Fig. 25** Microscopy images of TUNEL stained with the positive controls.

Supplementary **Fig. 26** Individual growth curves of 4T1 tumors.

Supplementary **Fig. 27** The body weight variation of 4T1 tumor-bearing mice.

Supplementary **Fig. 28** Individual growth curves of CT26 tumors.

Supplementary **Fig. 29** Photos of the representative CT26 tumor-bearing mice.

Supplementary **Fig. 30** The body weight variation of CT26 tumor-bearing mice.

Supplementary **Fig. 31** Individual growth curves of PDX tumors.

Supplementary **Fig. 32** The body weight variation of PDX tumor-bearing mice.

Supplementary **Fig. 33** Individual growth curves of VX2 tumors.

Supplementary **Fig. 34** The hematology profiles of mice implanted with MgG rods.

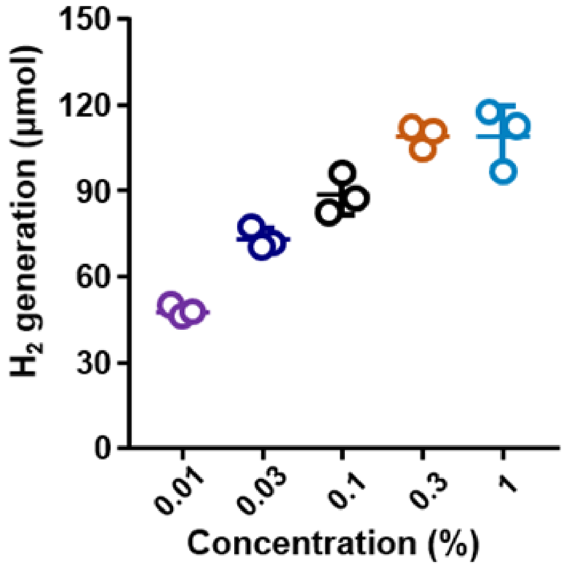

Supplementary Fig. 1. H_2 generation in 24 h from MgG rods prepared under different $PtCl_6^{2-}$ concentrations as measured by gas chromatography (n = 3 biologically independent samples). Data are presented as mean values ±SD.

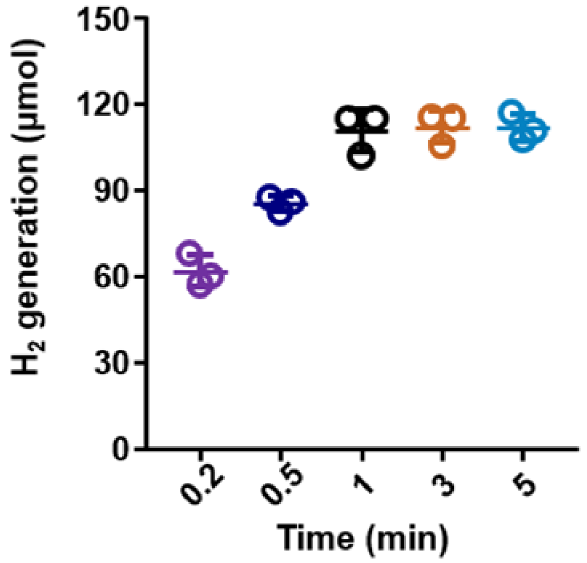

Supplementary Fig. 2. H_2 generation in 24 h from MgG rods prepared under different immersion time as measured by gas chromatography (n = 3 biologically independent samples). Data are presented as mean values ±SD.

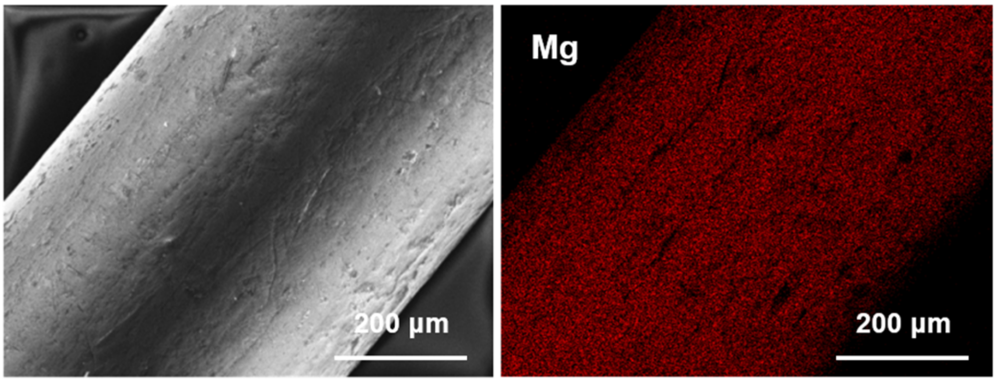

Supplementary Fig. 3. SEM and EDS mapping images of Mg rods. A representative image of three biologically independent samples.

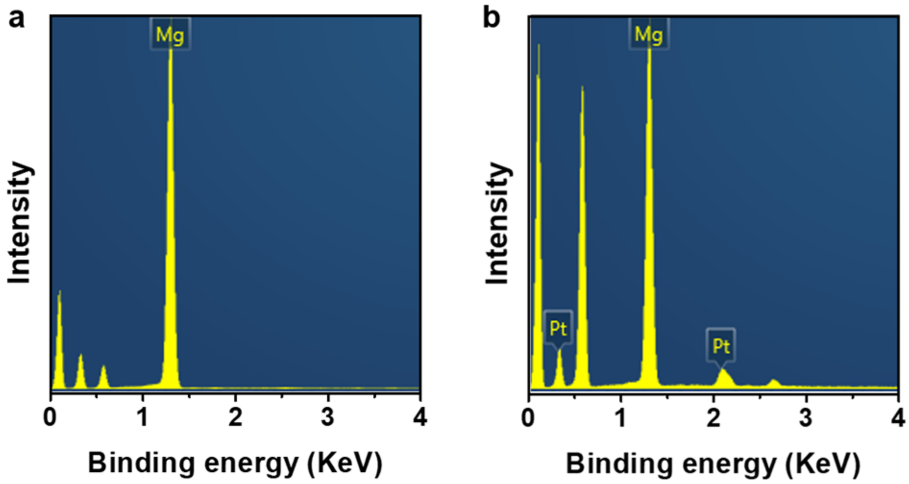

Supplementary Fig. 4. EDS spectra of Mg (**a**) and MgG (**b**).

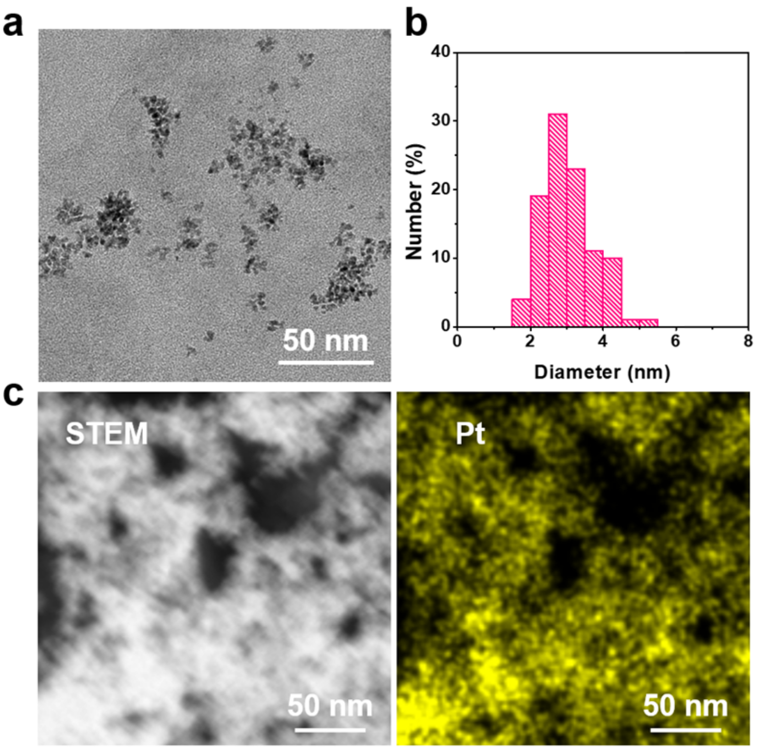

Supplementary Fig. 5. Characterization of Pt nanoparticles (NPs). (**a**) TEM image of Pt NPs. (**b**) Particle-size distribution (PSD) of Pt NPs determined by TEM image. (**c**) High-angle annular dark-field scanning TEM (HAADF-STEM) image and elemental mapping of Pt NPs. A representative image of three biologically independent samples is shown in Fig. S5a, c.

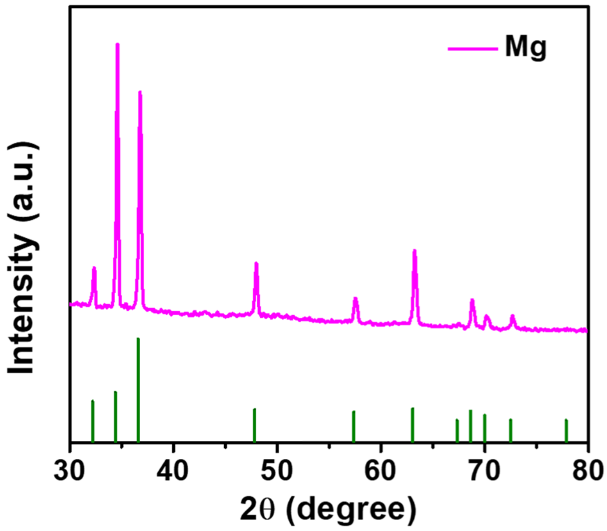

Supplementary Fig. 6. XRD sprectrum of Mg rods.

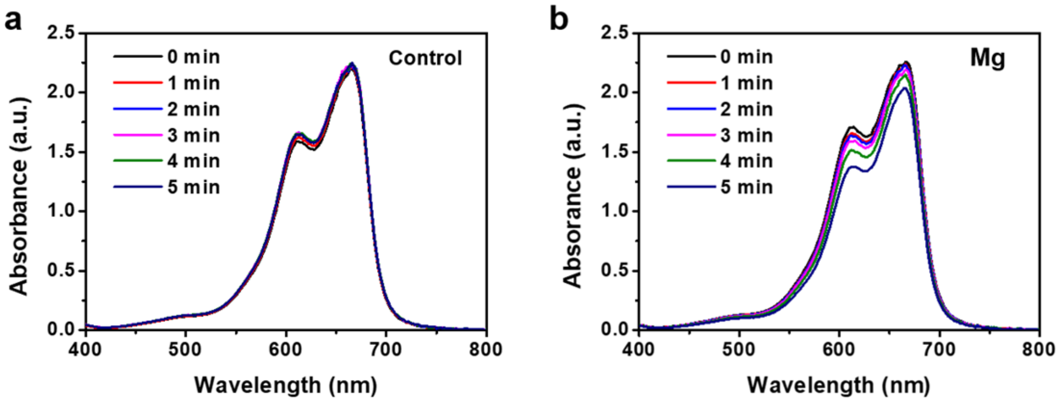

Supplementary Fig. 7. Time-dependent absorption spectra of the MB solution (pH = 6.5) via various treatments, including control (**a**) and Mg rods (**b**).

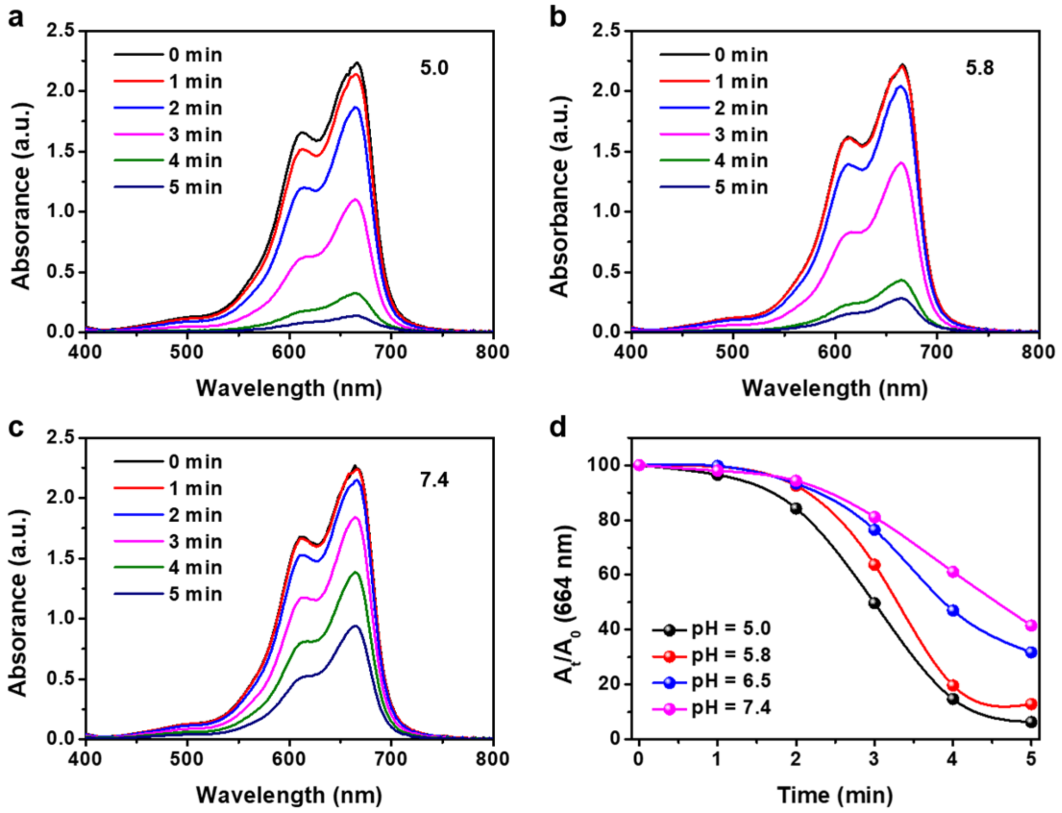

Supplementary Fig. 8. (**a-c**) Time-dependent absorption spectra of the MB solution with different pH values treated with MgG rods. (**d**) Comparison of MB reduction in PBS solutions with different pH values by MgG rods.

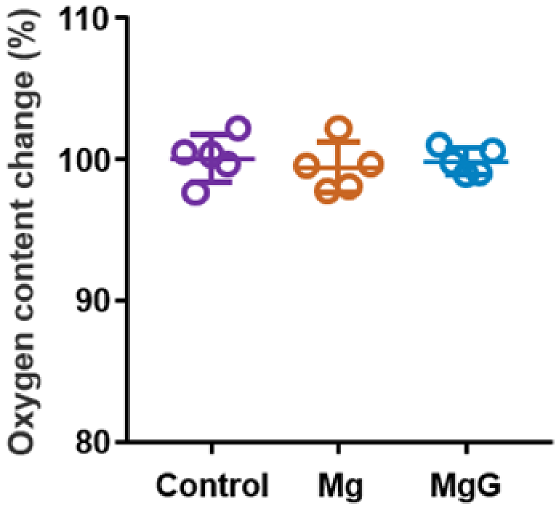

Supplementary Fig. 9. O_2 content changes after the reaction between Mg or MgG rods and H_2O measured by gas chromatography (n=5 biologically independent samples). Data are presented as mean values ±SD.

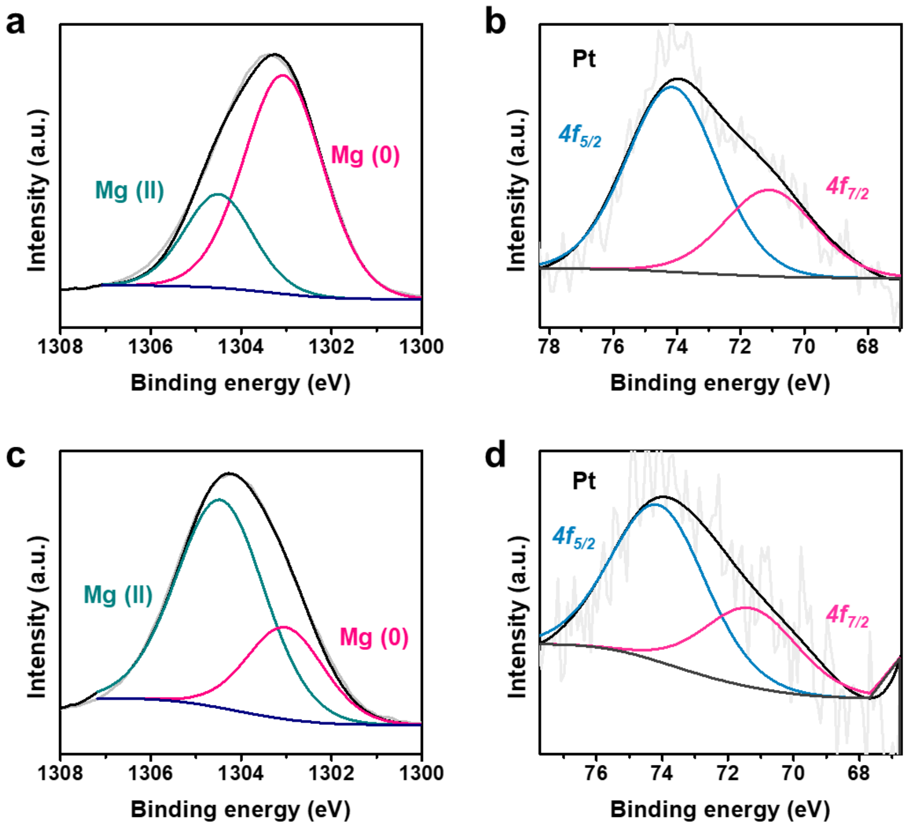

Supplementary Fig. 10. XPS characterization of MgG before (**a, b**) and after (**c, d**) H_2 generation process.

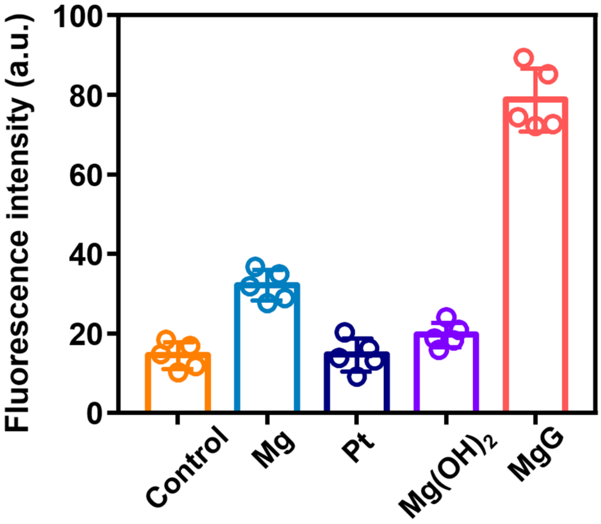

Supplementary Fig. 11. Quantitative analysis of the fluorescence intensity of JC-1 monomers in 4T1 cells measured by confocal fluorescence images (n = 5 biologically independent samples). Data are presented as mean values ±SD.

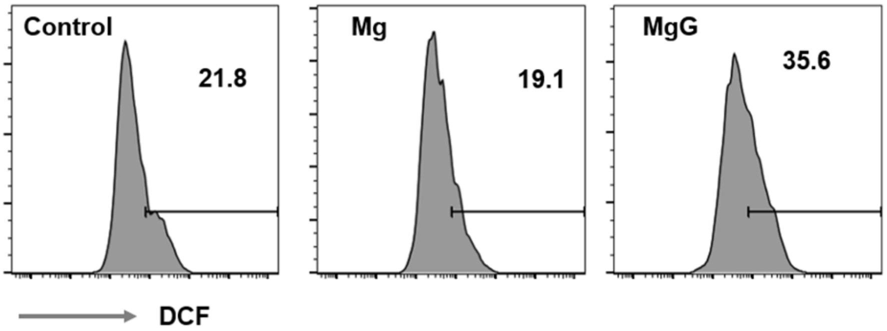

Supplementary Fig. 12. Flow cytometry data to show DCF-positive CT26 cells after different treatments.

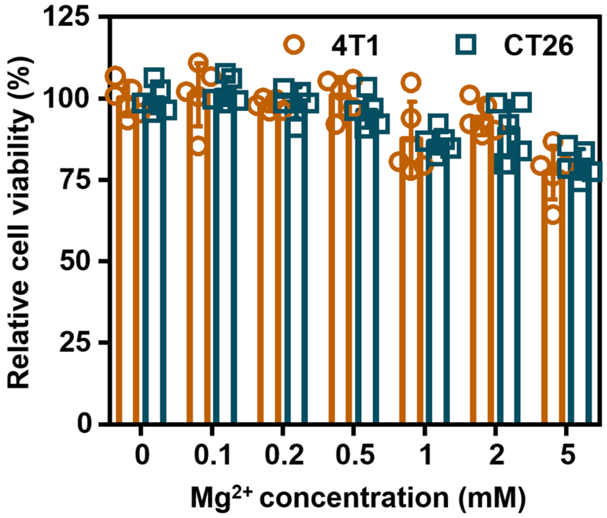

Supplementary Fig. 13. Relative cell viabilities of 4T1 and CT26 cells after incubation with various Mg^{2+} concentrations for 24 h (n = 5 biologically independent samples). Data are presented as mean values ±SD.

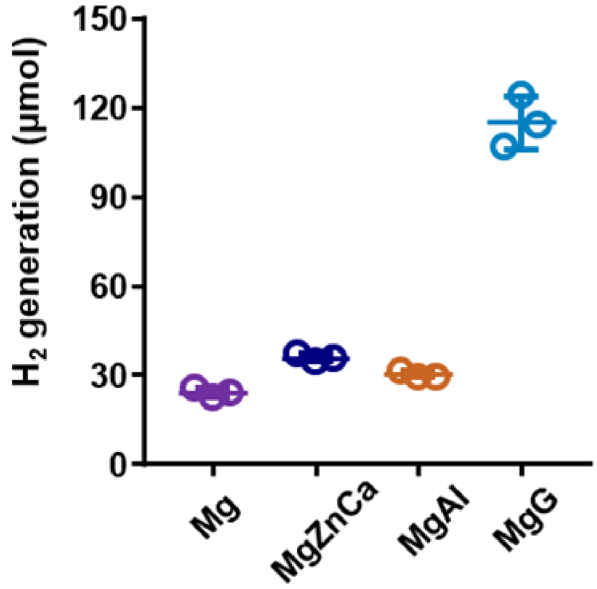

Supplementary Fig. 14. H_2 generation performance of MgG rods and different kinds of the commercialized Mg alloys (Mg, MgZnCa, and MgAl) measured by gas chromatography (n = 3 biologically independent samples). Data are presented as mean values ±SD.

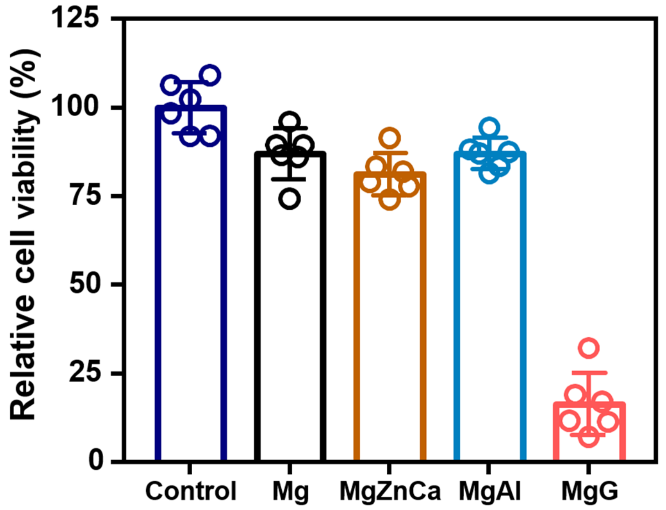

Supplementary Fig. 15. Relative viabilities of 4T1 cells after various treatments (Control, Mg, MgZnCa, MgAl, and MgG, n = 6 biologically independent samples). Data are presented as mean values ±SD.

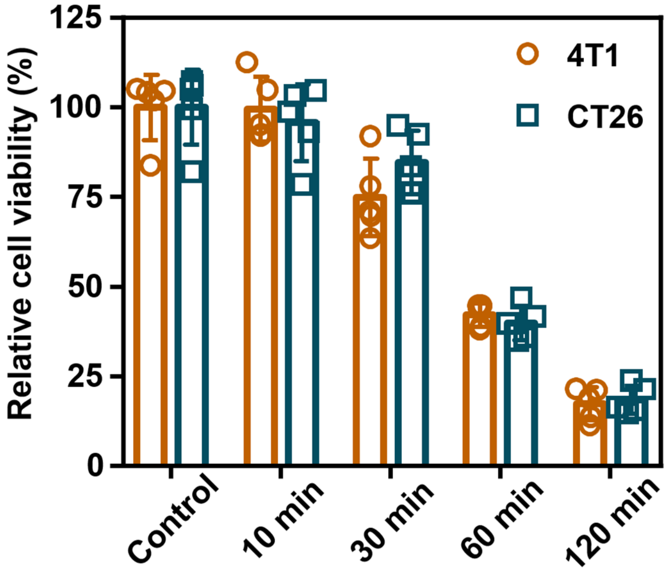

Supplementary Fig. 16. Cell viabilities of 4T1 and CT26 cells cultured with the pure H_2 released from a hydrogen balloon for different times (n = 5 biologically independent samples). Data are presented as mean values ±SD.

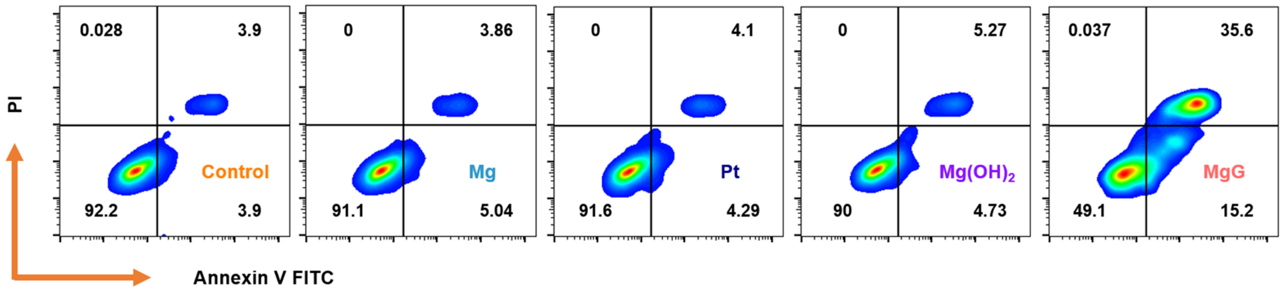

Supplementary Fig. 17. Flow cytometry analysis of CT26 cells after various treatments using an Annexin V-FITC/PI kit.

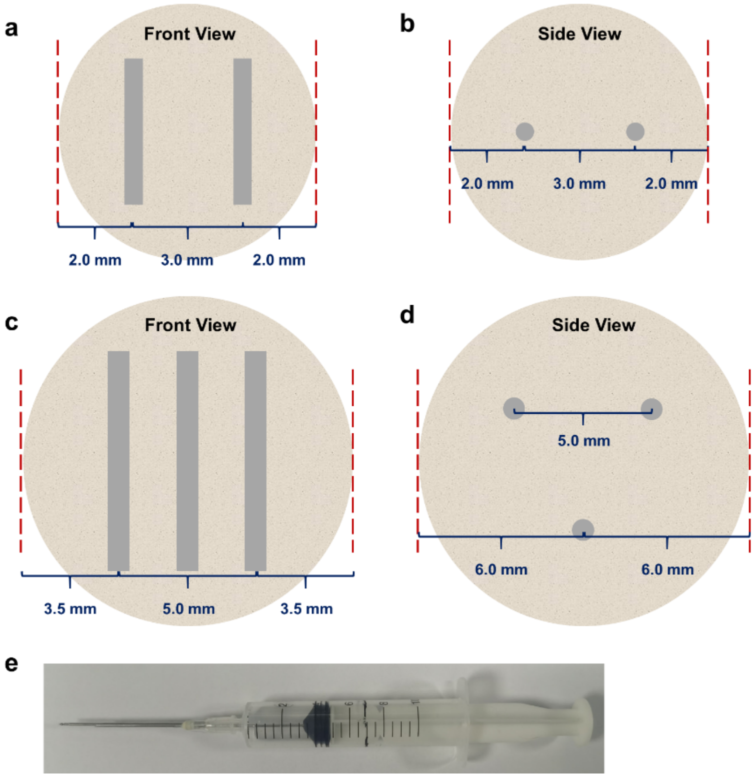

Supplementary Fig. 18. Schematic illustration the position of Mg or MgG rods in the tumors of mice (**a, b**) or rabbits (**c, d**) implanted by a simple implant approach (**e**).

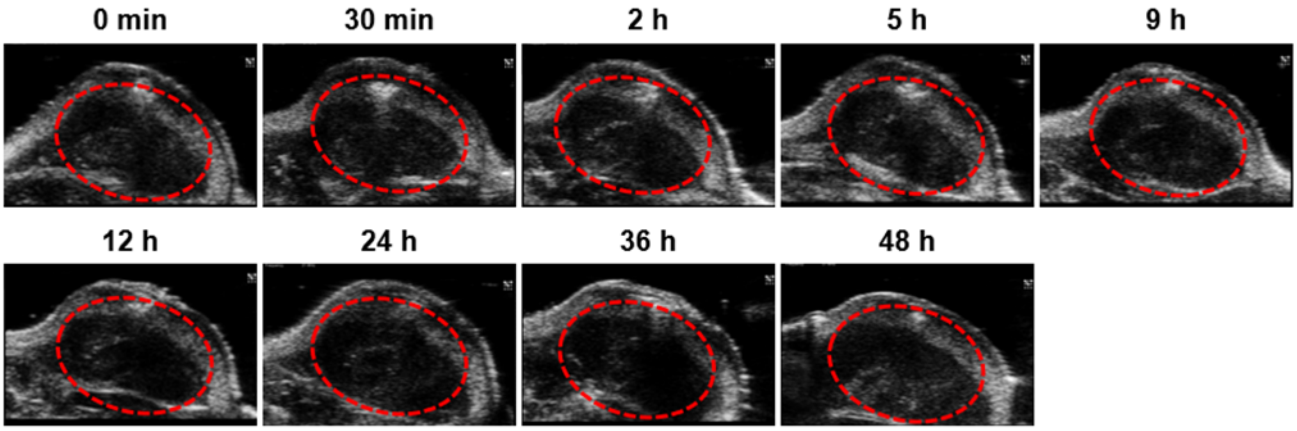

Supplementary Fig. 19. In vivo time-dependent ultrasonic imaging of 4T1 tumor-bearing mice post i.t. implanted with Mg rods. A representative image of three biologically independent samples.

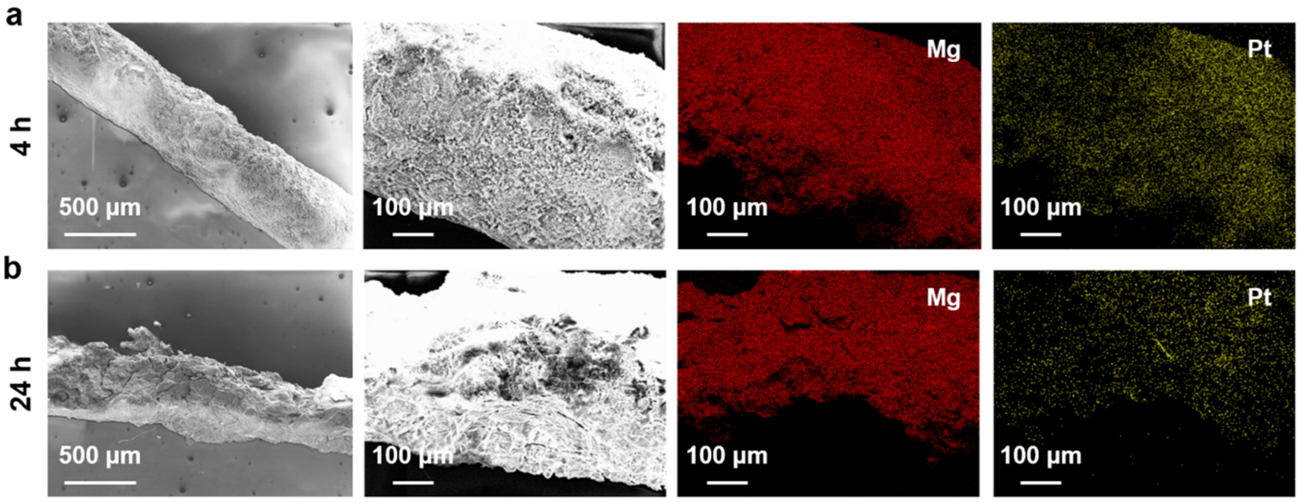

Supplementary Fig. 20. SEM image and EDS element mapping of MgG rods after implantation into the tumor for 4 h (**a**) and 24 h (**b**). A representative image of three biologically independent samples.

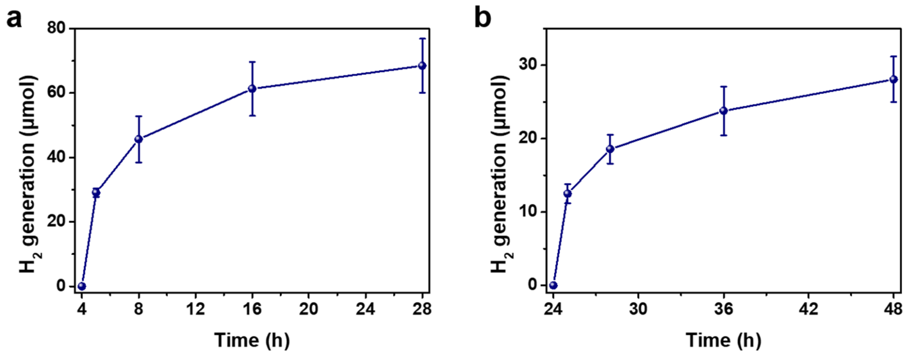

Supplementary Fig. 21. H_2 generation from MgG rods in PBS solutions after implantation into the tumor for 4 (**a**) and 24 hours (**b**) as measured by gas chromatography. (n = 3 biologically independent samples). Data are presented as mean values ±SD.

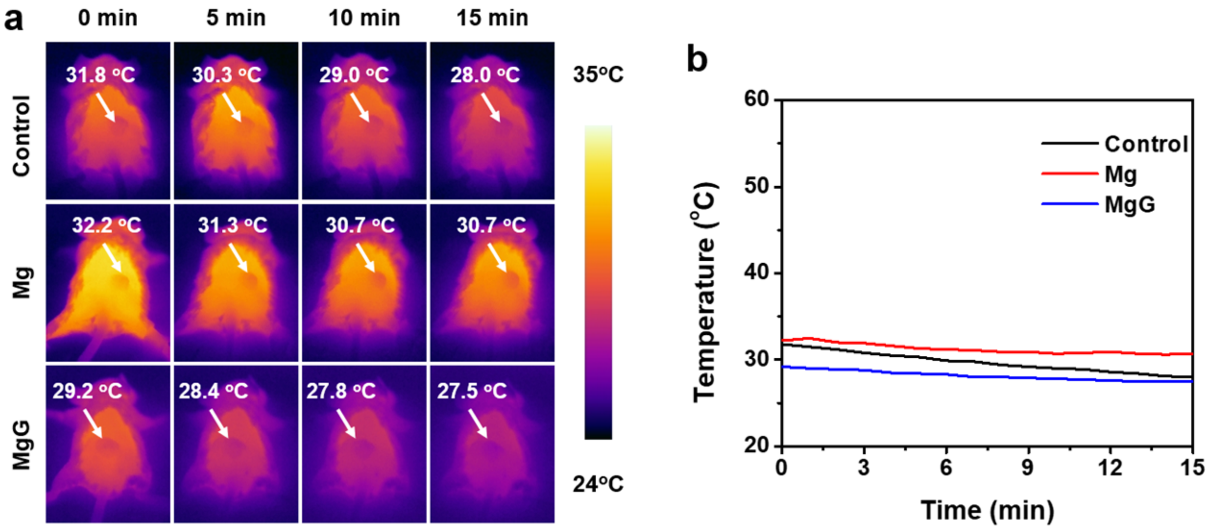

Supplementary Fig. 22. The infrared thermal images (**a**) and temperature change curves (**b**) of mice tumor at different time points via various treatments.

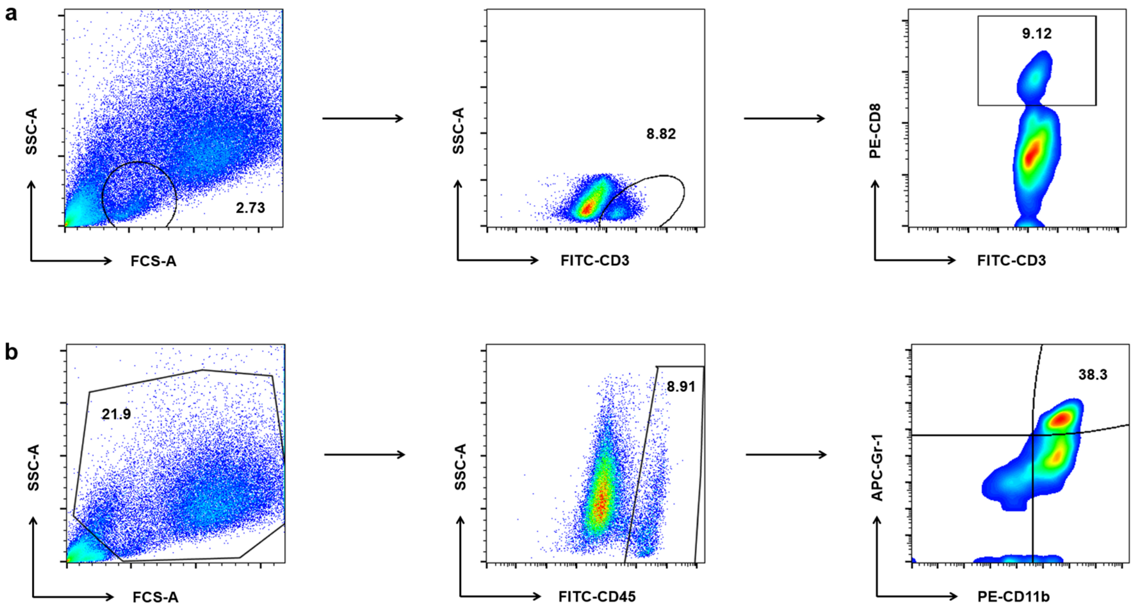

Supplementary Fig. 23. Flow cytometry gating strategy for analysis of different cells in tumor. (**a**) T cells ($CD3^+$) and $CD8^+$ T cells ($CD3^+CD8^+$). (**b**) myeloid-derived suppressor cells (MDSCs, $CD45^+CD11b^+Gr\text{-}1^+$).

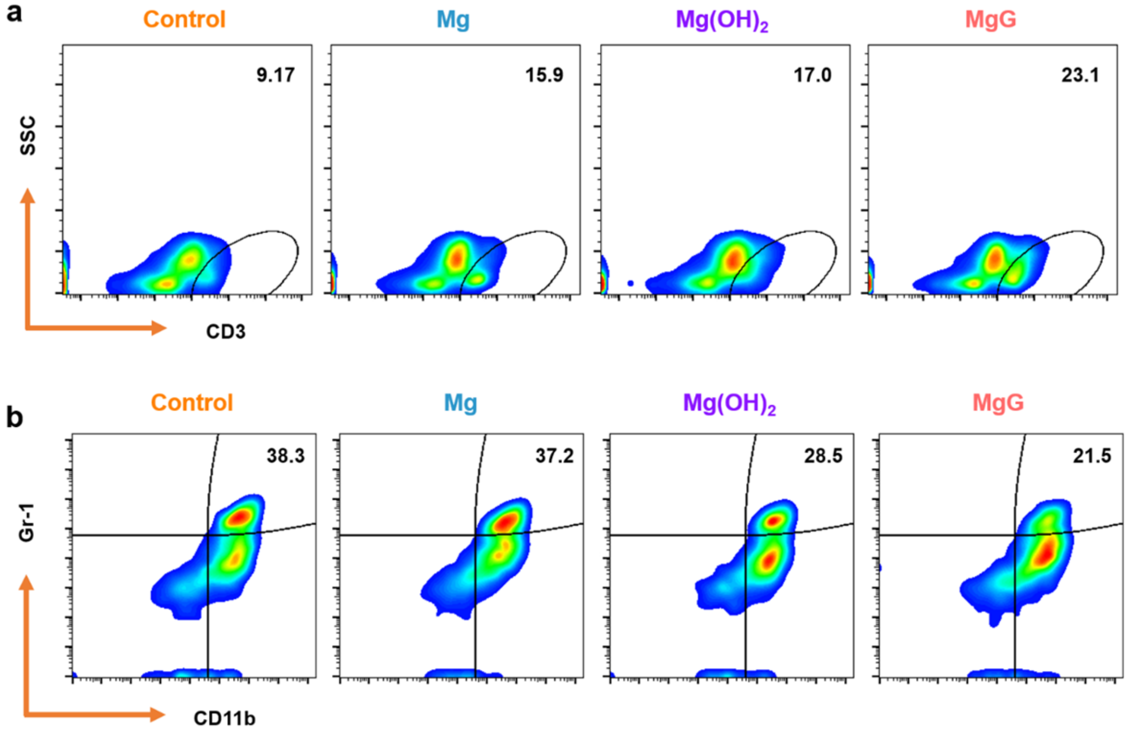

Supplementary Fig. 24. The flow cytometric analysis results of T cells (**a**, $CD3^+$) and MDSCs (**b**, $CD45^+CD11b^+Gr\text{-}1^+$) within the tumors after different treatments.

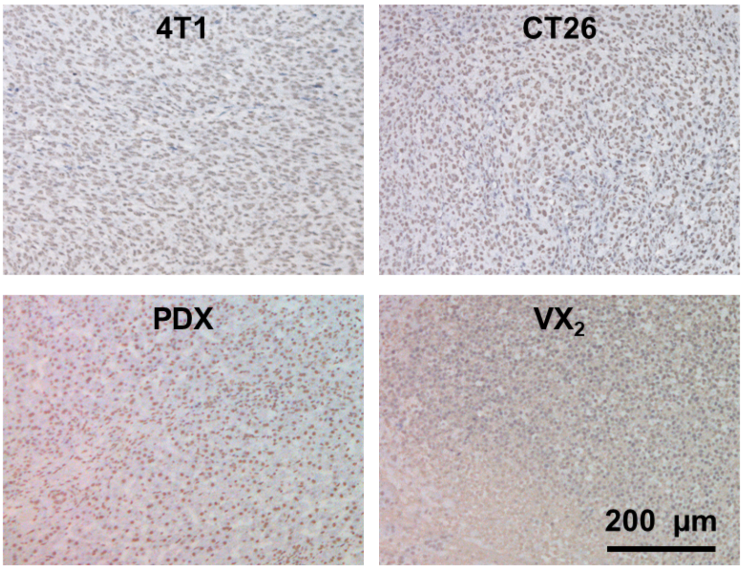

Supplementary Fig. 25. Microscopy images of TUNEL stained with the positive controls in different tumor sections. A representative image of three biologically independent animals.

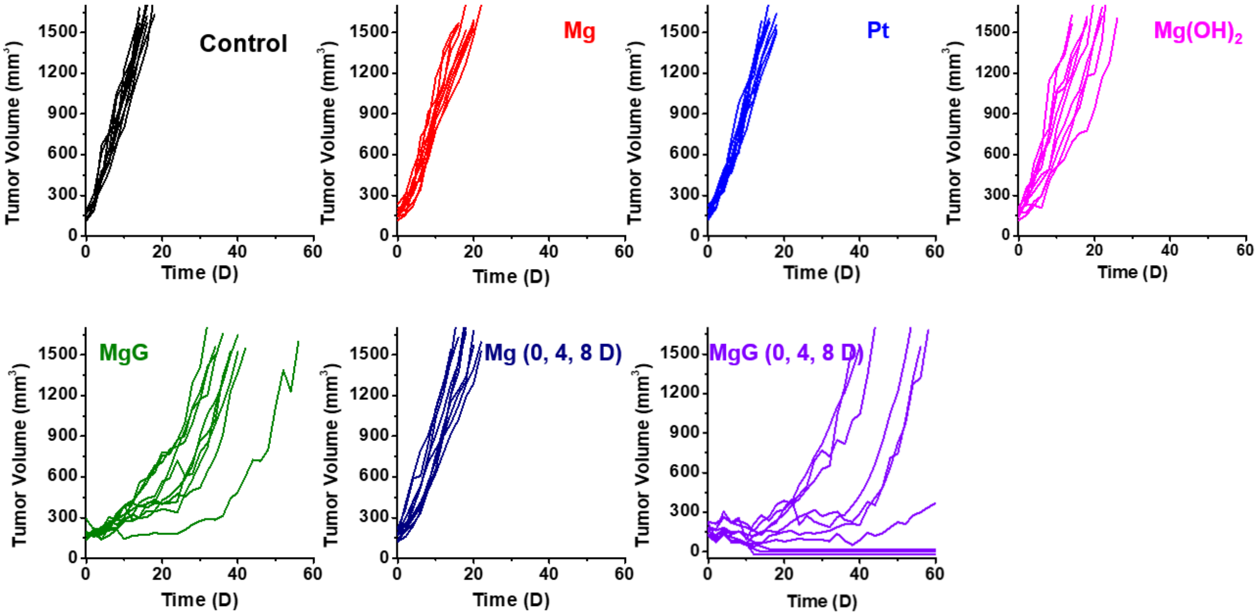

Supplementary Fig. 26. Individual growth curves of 4T1 tumors after various treatments.

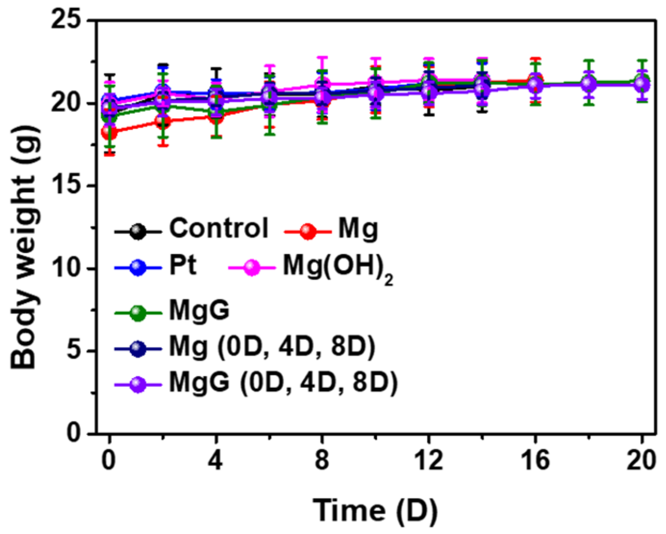

Supplementary Fig. 27. The body weight variation of 4T1 tumor-bearing mice post various treatments (n = 10 biologically independent animals). Data are presented as mean values ±SD.

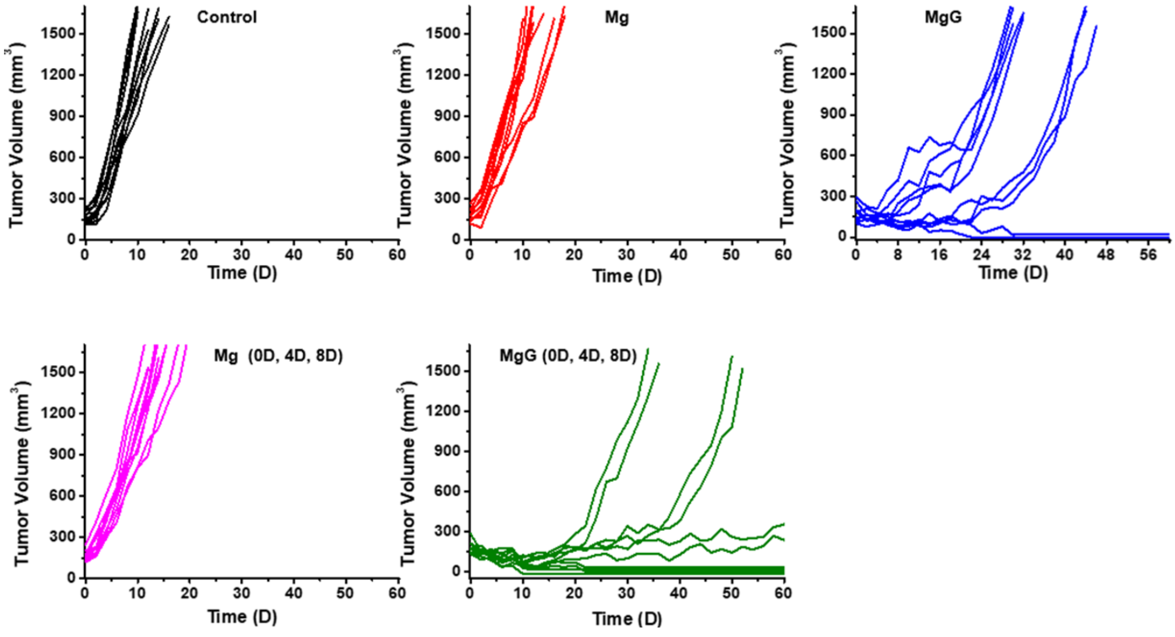

Supplementary Fig. 28. Individual growth curves of CT26 tumors after various treatments.

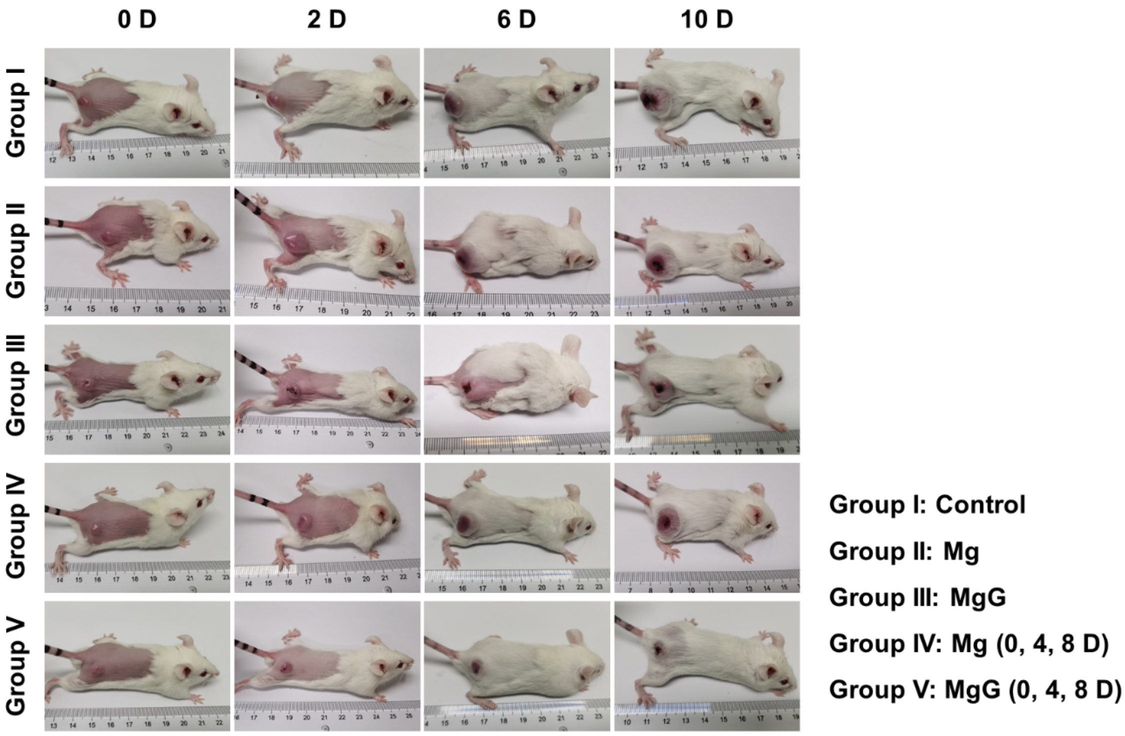

Supplementary Fig. 29. Photos of the representative CT26 tumor-bearing mice taken at different days after various treatments.

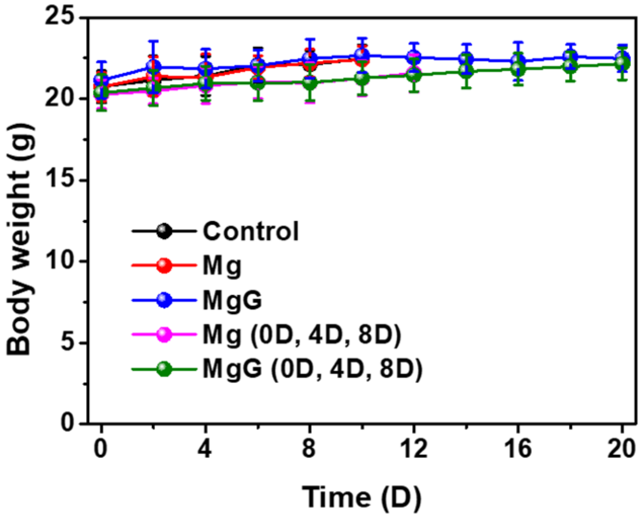

Supplementary Fig. 30. The body weight variation of CT26 tumor-bearing mice post various treatments (n = 10 biologically independent animals). Data are presented as mean values ±SD.

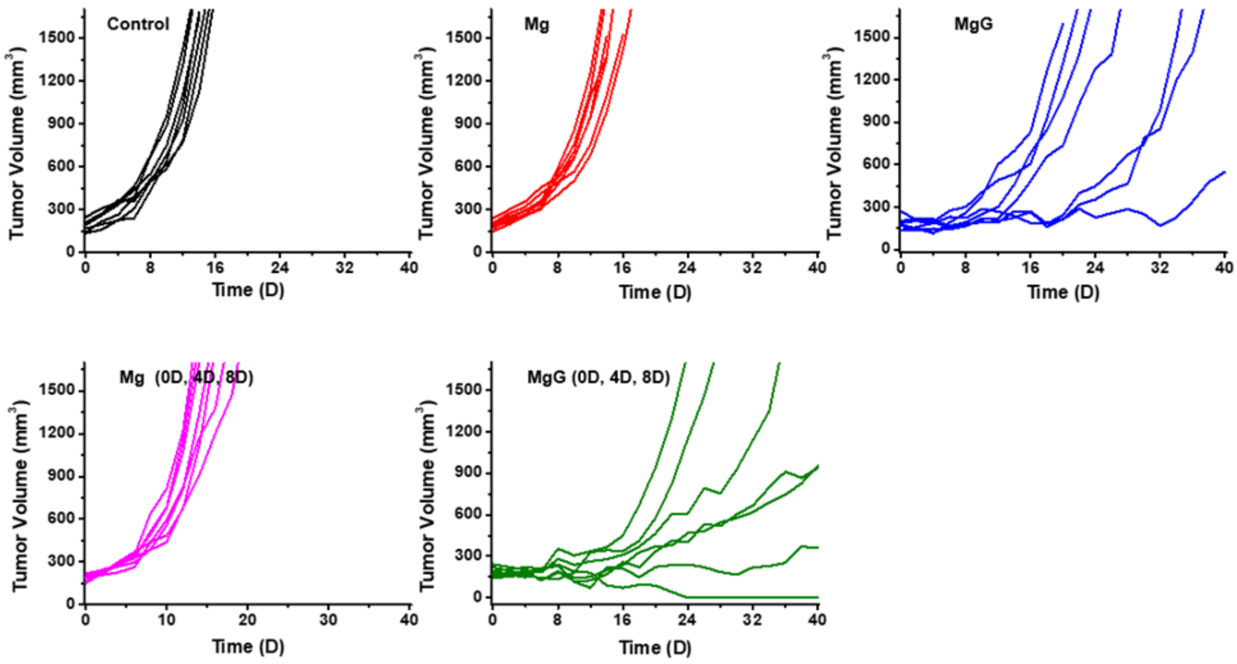

Supplementary Fig. 31. Individual growth curves of PDX tumors after various treatments.

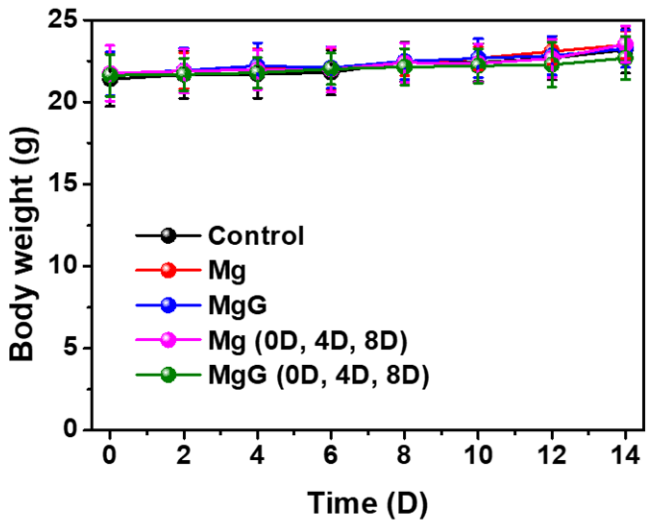

Supplementary Fig. 32. The body weight variation of PDX tumor-bearing mice post various treatments (n = 7 biologically independent animals). Data are presented as mean values ±SD.

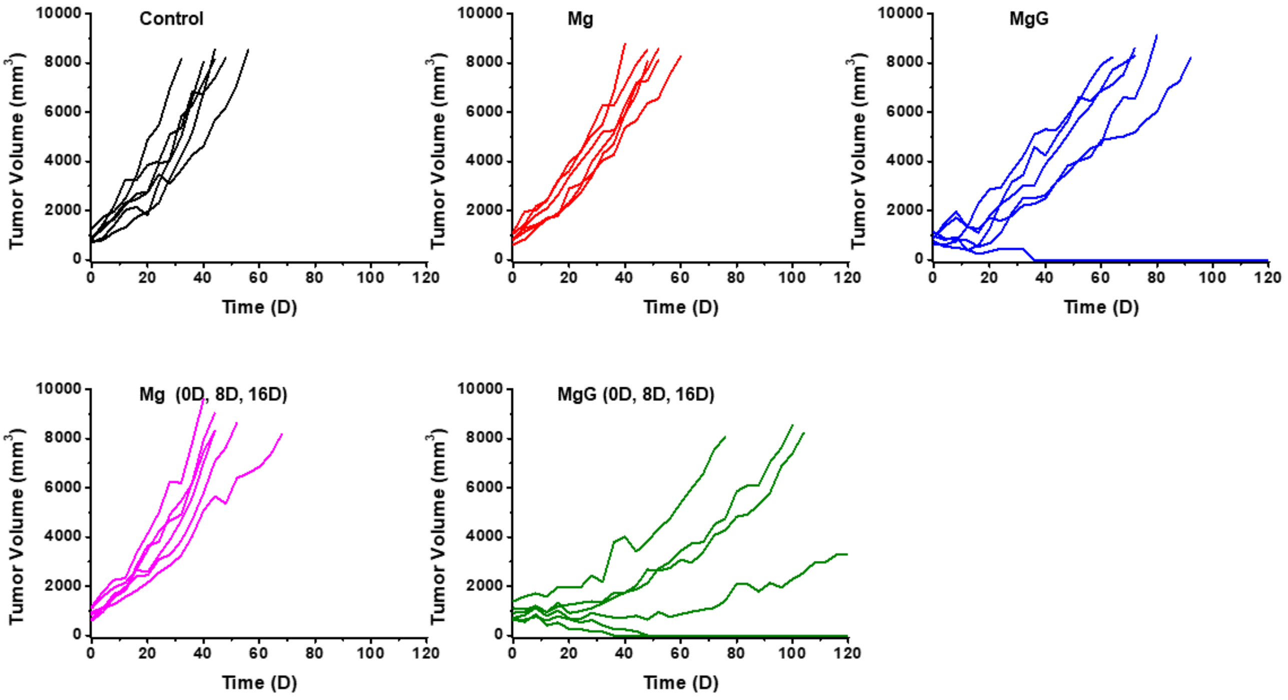

Supplementary Fig. 33. Individual growth curves of VX_2 tumors after various treatments.

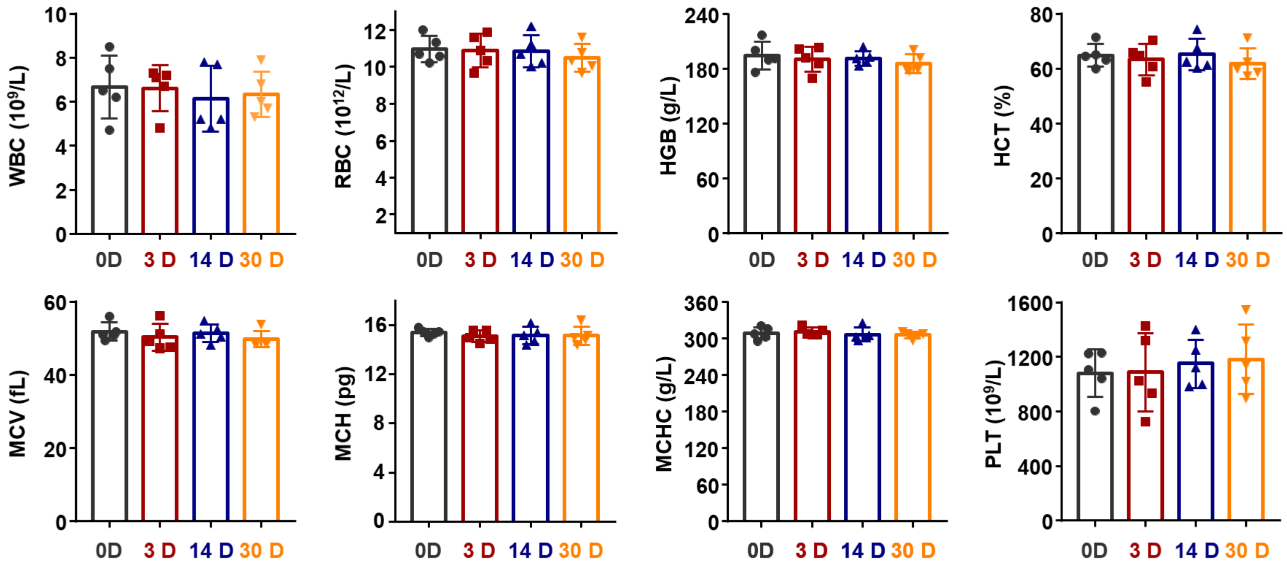

Supplementary Fig. 34. The hematology profiles of mice implanted with MgG rods (D = 0.5 mm, L = 4.0 mm, two rods per mouse) at various time points (0, 3, 14, and 30 D). n = 5 biologically independent animals. Data are presented as mean values ±SD.
