# Supplementary material for: Magnesium galvanic cells produce hydrogen and modulate the tumor microenvironment to inhibit cancer growth
Source: Nat Commun. 2022 Apr 28;13:2336. doi: 10.1038/s41467-022-29938-6 (PMC9051066; doi:10.1038/s41467-022-29938-6)
Supplement: Supplementary file 4 — Reporting Summary [file 41467_2022_29938_MOESM4_ESM.pdf]

nature portfolio

Corresponding author(s): Liang Cheng
Last updated by author(s): Apr 2, 2022

Reporting Summary

Nature Portfolio wishes to improve the reproducibility of the work that we publish. This form provides structure for consistency and transparency in reporting. For further information on Nature Portfolio policies, see our Editorial Policies and the Editorial Policy Checklist.

Statistics

For all statistical analyses, confirm that the following items are present in the figure legend, table legend, main text, or Methods section.

n/a	Confirmed	
☐	☒	The exact sample size (*n*) for each experimental group/condition, given as a discrete number and unit of measurement
☐	☒	A statement on whether measurements were taken from distinct samples or whether the same sample was measured repeatedly
☐	☒	The statistical test(s) used AND whether they are one- or two-sided<br>*Only common tests should be described solely by name; describe more complex techniques in the Methods section.*
☐	☒	A description of all covariates tested
☐	☒	A description of any assumptions or corrections, such as tests of normality and adjustment for multiple comparisons
☐	☒	A full description of the statistical parameters including central tendency (e.g. means) or other basic estimates (e.g. regression coefficient) AND variation (e.g. standard deviation) or associated estimates of uncertainty (e.g. confidence intervals)
☐	☒	For null hypothesis testing, the test statistic (e.g. *F*, *t*, *r*) with confidence intervals, effect sizes, degrees of freedom and *P* value noted<br>*Give P values as exact values whenever suitable.*
☒	☐	For Bayesian analysis, information on the choice of priors and Markov chain Monte Carlo settings
☒	☐	For hierarchical and complex designs, identification of the appropriate level for tests and full reporting of outcomes
☒	☐	Estimates of effect sizes (e.g. Cohen's *d*, Pearson's *r*), indicating how they were calculated

Our web collection on statistics for biologists contains articles on many of the points above.

Software and code

Policy information about availability of computer code

Data collection	Scanning electron microscope (SEM), X-ray diffractometer (XRD), UV-vis-NIR spectrophotometer, gas chromatography, electrochemical analyzer, Inductively Coupled Plasma Optical Emission Spectrometry (ICP-OES), Infrared Camera, Confocal Microscope, ultrasonic imaging system, pH microsensor, optical imaging system, ChemDraw 2014, Excel 2019, Origin 8.5, ImageJ (version 6), FlowJo (version 10.4).
Data analysis	All statistical analyses were performed on Excel 2019, Origin 8.5 and Graphpad Prism 7.

For manuscripts utilizing custom algorithms or software that are central to the research but not yet described in published literature, software must be made available to editors and reviewers. We strongly encourage code deposition in a community repository (e.g. GitHub). See the Nature Portfolio guidelines for submitting code & software for further information.

Data

Policy information about availability of data

All manuscripts must include a data availability statement. This statement should provide the following information, where applicable:

- Accession codes, unique identifiers, or web links for publicly available datasets
- A description of any restrictions on data availability
- For clinical datasets or third party data, please ensure that the statement adheres to our policy

All the data supporting the findings of this study are available within the article and its Supplementary Information file and from the corresponding authors upon reasonable request.

Field-specific reporting

Please select the one below that is the best fit for your research. If you are not sure, read the appropriate sections before making your selection.

☒ Life sciences ☐ Behavioural & social sciences ☐ Ecological, evolutionary & environmental sciences

For a reference copy of the document with all sections, see nature.com/documents/nr-reporting-summary-flat.pdf

Life sciences study design

All studies must disclose on these points even when the disclosure is negative.

Sample size	No statistical methods were used to predetermine the sample sizes. It is impossible to predict the magnitude of experimental variation between animals based on our current knowledge. The group sizes (at least three animals per treatment group) represents the minimum number animals needed to reach statistical significance ($p < 0.05$) between experimental groups.
Data exclusions	No data were excluded.
Replication	Experiments were repeated and experimental findings were reproducible. All experiments were run at least in triplicate.
Randomization	All experimental samples or animals (mice and rabbits) were allocated randomly to each group.
Blinding	Mice and rabbits were used in this work. Blinding and randomization were applied to all experiments.

Reporting for specific materials, systems and methods

We require information from authors about some types of materials, experimental systems and methods used in many studies. Here, indicate whether each material, system or method listed is relevant to your study. If you are not sure if a list item applies to your research, read the appropriate section before selecting a response.

Materials & experimental systems

n/a	Involved in the study	
☐	☒	Antibodies
☐	☒	Eukaryotic cell lines
☒	☐	Palaeontology and archaeology
☐	☒	Animals and other organisms
☐	☒	Human research participants
☒	☐	Clinical data
☒	☐	Dual use research of concern

Methods

n/a	Involved in the study	
☒	☐	ChIP-seq
☐	☒	Flow cytometry
☒	☐	MRI-based neuroimaging

Antibodies

Antibodies used	T cells: anti-CD3-FITC (Biolegend, clone 17A2, Catalog: 100204), anti-CD8-PE (Biolegend, clone 53-6.7, Catalog: 100708), anti-CD45-FITC (Biolegend, clone I3/2.3, Catalog: 147710), anti-CD4-APC (Biolegend, clone GK1.5, Catalog: 100412).<br>MDSCs cells: anti-CD11b-PE (Biolegend, clone M1/70, Catalog: 101208), anti-Gr-1-APC (Biolegend, clone RB6-8C5, Catalog: 108412), anti-CD45-FITC (Biolegend, clone I3/2.3, Catalog: 147710).
Validation	All antibodies were verified by the supplier and each lot has been quality tested. All validation statements can be found on the respective antibody website:<br>1. Anti-mouse-CD3-FITC: https://www.biolegend.com/en-us/products/fitc-anti-mouse-cd3-antibody-45<br>2. Anti-mouse-CD8a-PE: https://www.biolegend.com/en-us/products/pe-anti-mouse-cd8a-antibody-155?Clone=53-6.7<br>3. Anti-mouse-CD11b-PE: https://www.biolegend.com/en-us/search-results/pe-anti-mouse-human-cd11b-antibody-349<br>4. Anti-mouse-Gr-1-APC: https://www.biolegend.com/en-us/search-results/apc-anti-mouse-ly-6g-ly-6c-gr-1-antibody-456<br>5. Anti-mouse-CD45-FITC: https://www.biolegend.com/en-us/products/fitc-anti-mouse-cd45-antibody-9796?Clone=I3/2.3<br>6. Anti-mouse-CD4-APC: https://www.biolegend.com/en-us/products/apc-anti-mouse-cd4-antibody-245

Eukaryotic cell lines

Policy information about cell lines

Cell line source(s)	4T1 murine breast cancer cells (SCSP-5056) and CT26 colon cancer cells (TCM37) were obtained from the Cell Bank, Shanghai Institutes for Biological Sciences, Chinese Academy of Sciences. Luciferase-transfected 4T1 cells (Luc-4T1) was obtained from PerkinElmer Co. as a gift, Rabbit VX2 liver cancer cells (MZ-0769) was obtained from Ningbo Mingzhou Biological Technology Co., Ltd.

Authentication	Identity of the cell lines were frequently checked by their morphological features but have not been authenticated by the short tandem repeat (STR) profiling.
Mycoplasma contamination	All cell lines were tested for mycoplasma contamination. No mycoplasma contamination was found.
Commonly misidentified lines (See ICLAC register)	No commonly misidentified cell lines were used.

Animals and other organisms

Policy information about studies involving animals; ARRIVE guidelines recommended for reporting animal research

Laboratory animals	Female Balb/c mice (6–8 weeks), female Balb/c nude mice (6–8 weeks) and New Zealand white rabbits (3–5 months) were used as animal model in this work. Mice were housed in groups of 5 mice per individually ventilated cage in a 12-h light-dark cycle (8:00–20:00 light; 20:00–8:00 dark), with constant room temperature (21 ± 1 °C) and relative humidity (40-70%). All mice had access to food and water ad libitum.
Wild animals	The study did not involve wild animals.
Field-collected samples	The study did not involve samples collected from field.
Ethics oversight	The experimental protocols were approved by the Institutional Animal Care and Use Committee (IACUC) of the Animal Experiment Center of Soochow University (Suzhou, China, No. ECSU-2020000175). All animal experimental procedures were performed in accordance with the Regulations for the Administration of Affairs Concerning Experimental Animals approved by the State Council of the People's Republic of China.

Note that full information on the approval of the study protocol must also be provided in the manuscript.

Human research participants

Policy information about studies involving human research participants

Population characteristics	For the patient-derived xenograft tumor model (PDX), patients' cervical tumor tissue was acquired from middle-aged female patient with cervical cancer in Suzhou Municipal Hospital after obtaining informed consent.
Recruitment	Patients' cervical tumor tissue was acquired from patient who seek medical care independently with cervical cancer in the First Affiliated Hospital of Soochow University after obtaining informed consent. H&E and TUNEL staining of patients' cervical tumor tissue section was used to demonstrate the viability.
Ethics oversight	Patients' cervical tumor tissue was used following protocols approved by the First Affiliated Hospital of Soochow University (No. HESU-2020001032)

Note that full information on the approval of the study protocol must also be provided in the manuscript.

Flow Cytometry

Plots

Confirm that:

☒ The axis labels state the marker and fluorochrome used (e.g. CD4-FITC).

☒ The axis scales are clearly visible. Include numbers along axes only for bottom left plot of group (a 'group' is an analysis of identical markers).

☒ All plots are contour plots with outliers or pseudocolor plots.

☒ A numerical value for number of cells or percentage (with statistics) is provided.

Methodology

Sample preparation	The tumor tissue samples were surgically removed from the mice, and then processed through mechanical disruption before digestion for 30 min at 37 °C in RPMI-1640 (10% heat-inactivated FBS and 1% PS) containing 1.5mg/mL collagenase IV(Sigma), 1.5mg/mL collagenase I (Sigma), 1.5 mg/mL hyaluronidase (Sigma), and 0.2 mg/mL DNase I (Sigma). The samples were then passed through 200-mesh nylon mesh filters to obtain single-cell suspensions.<br>For all samples, cells were stained with antibodies against surface antigens.
Instrument	BD AccuritTM C6 Plus
Software	FlowJo software package (version 10.4)
Cell population abundance	No sorting was performed
Gating strategy	Gating strategies are referred to those described in the website (https://www.bio-rad-antibodies.com/blog/a-guide-togating-in-flow-cytometry.html). In general, cells were first gated on FSC/SSC. Surface and intracellular antigen gating was performed

on the first gating cell population. The cell populations within the gate were further analysised based on expression of markers. Single positive straining were uesd to determing the "true" positive. Gating was then based on positive level (after proper compensation). The detailed gating strategy could be found in supporting information.

☒ Tick this box to confirm that a figure exemplifying the gating strategy is provided in the Supplementary Information.
